# Internet trials: participant experiences and perspectives

**DOI:** 10.1186/1471-2288-12-162

**Published:** 2012-10-23

**Authors:** Erin Mathieu, Alexandra Barratt, Stacy M Carter, Gro Jamtvedt

**Affiliations:** 1 School of Medicine, University of Western Sydney, Campbelltown, Australia; 2 School of Public Health, University of Sydney, Sydney, Australia; 3 Centre for Values, Ethics and Law in Medicine, University of Sydney, Sydney, Australia; 4 The Norwegian Knowledge Centre for the Health Services, St Olvas plass N-0130, Oslo, Norway

**Keywords:** Internet, Randomized controlled trials, Participant experience, Methods, Methodology

## Abstract

**Background:**

Use of the Internet to conduct randomised controlled trials is increasing, and provides potential to increase equity of access to medical research, increase the generalisability of trial results and decrease the costs involved in conducting large scale trials. Several studies have compared response rates, completeness of data, and reliability of surveys using the Internet and traditional methods, but very little is known about participants’ attitudes towards Internet-based randomised trials or their experience of participating in an Internet-based trial.

**Objective:**

To obtain insights into the experiences and perspectives of participants in an Internet-based randomised controlled trial, their attitudes to the use of the Internet to conduct medical research, and their intentions regarding future participation in Internet research.

**Methods:**

All English speaking participants in a recently completed Internet randomised controlled trial were invited to participate in an online survey.

**Results:**

1246 invitations were emailed. 416 participants completed the survey between May and October 2009 (33% response rate). Reasons given for participating in the Internet RCT fell into 4 main areas: personal interest in the research question and outcome, ease of participation, an appreciation of the importance of research and altruistic reasons. Participants’ comments and reflections on their experience of participating in a fully online trial were positive and less than half of participants would have participated in the trial had it been conducted using other means of data collection. However participants identified trade-offs between the benefits and downsides of participating in Internet-based trials. The main trade-off was between flexibility and convenience – a perceived benefit – and a lack connectedness and understanding – a perceived disadvantage. The other tradeoffs were in the areas of: ease or difficulty in use of the Internet; security, privacy and confidentiality issues; perceived benefits and disadvantages for researchers; technical aspects of using the Internet; and the impact of Internet data collection on information quality. Overall, more advantages were noted by participants, consistent with their preference for this mode of research over others. The majority of participants (69%) would prefer to participate in Internet-based research compared to other modes of data collection in the future.

**Conclusion:**

Participants in our survey would prefer to participate in Internet-based trials in the future compared to other ways of conducting trials. From the participants’ perspective, participating in Internet-based trials involves trade-offs. The central trade-off is between flexibility and convenience – a perceived benefit – and lack of connectedness and understanding – a perceived disadvantage. Strategies to maintain the convenience of the Internet while increasing opportunities for participants to feel supported, well-informed and well-understood would seem likely to increase the acceptability of Internet-based trials.

## Introduction

Since the 1950s, randomised controlled trials (RCTs) have been used to test the effects of health interventions [1]. RCTs have become the accepted gold standard for evaluating interventions and their now well accepted key principles ensure a minimization of bias which is more frequently observed in other types of studies [2]. A recent development in clinical trial methods has been the emergence of Internet-based RCTs. In Internet-based trials, the Internet is used for recruitment of trial participants, delivery of the health intervention, and/or data collection [3-5].

Internet-based trials have several potential advantages over conventional trials. Participants from all over the world can participate in a trial managed at a single site, decreasing costs and potentially increasing the equity of access to medical research. As it is possible to recruit large, diverse samples, including participants who would otherwise be difficult to access, Internet trials may also increase transferability of findings. This is an important advantage as conventional trials commonly recruit highly selected groups of participants, thus limiting the transferability of their results and the overall relevance and value of the research [6]. Finally, participants may feel more comfortable participating anonymously in Internet-based trials, and may be more open and honest with self-completed questionnaires [7].

Because of these advantages, use of Internet trials to test health interventions is increasing, and some of the methodological limitations are being explored [8-11]. These include higher rates of loss to follow-up than conventional trials, the uncertainty surrounding compliance with the intervention and the possibility of bias arising from self-reported outcomes. Generalisability may also be compromised by the exclusion of people who do not have access to the Internet. Several studies have compared response rates, completeness of data, and reliability of *surveys* using the Internet and traditional methods. However very little is known about participants’ attitudes towards Internet-based *trials* or about their experience of participating in an Internet- based trial. This could be important because better understanding may facilitate recruitment of participants to Internet trials and suggest ways in which follow-up rates and the outcome reporting may be improved, thus addressing the currently recognised limitations.

Our aims were to obtain insights into the experiences and perspectives of participants in an Internet-based RCT, describe their attitudes to the use of the Internet to conduct medical research, and explore their intentions and preferences regarding future participation in research. We also took the opportunity to enquire about people’s willingness to make false statements when participating in online (and other modes of) research.

## Methods

We conducted an online survey of participants in a recent Internet-based RCT [12]. The University of Sydney Human Research Ethics Committee approved the study.

### Setting and participants

All English speaking participants in a recently completed international Internet RCT (The Stretching Trial) were invited by email to participate in the survey. The Stretching Trial has been described elsewhere: it sought to test the efficacy of stretching to reduce injury or soreness after exercise [12]. Briefly, the trial was conducted between January 2008 and February 2009. Participation was open to people anywhere in the world who satisfied the following criteria: aged 18 years or over, able to read and write in English or Norwegian, taken part in vigorous physical activity on at least 1 day in the past week, and regularly access the Internet and email. The study was advertised via several channels including a radio programme in Australia, email messages and through a television programme in Norway. Potential participants visited the trial website and were screened for eligibility, and were randomised to stretch seven muscle groups on each side of the body (lower limb and trunk) before and after every occasion of physical activity for 12 weeks or not to stretch any lower limb or trunk muscles over the 12 week period. Participants returned to the trial website each week for 12 weeks to report on their injuries and soreness for the past week. Participants were sent weekly emails with links to the website and a reminder email was sent 3 days later if the participant had not completed their report.

### Data collection

Invitations to participate in this survey were emailed to the registered email address of participants who completed The Stretching Trial in English. Embedded within this invitation was a unique link that provided participants with password-protected access to the online survey. Participants who did not complete the survey within 6 weeks were emailed one reminder invitation to participate.

The survey sought to elicit information from participants regarding their experience of participation in The Stretching Trial, their thoughts on Internet trials and their preferences in relation to participating in Internet versus conventional trials. As we could not find any published surveys that adequately captured the information we desired, survey questions were designed specifically for this study.

There were two different versions of the survey. The standard survey consisted of a maximum of 41 questions, of which 17 were conditional on responses to previous questions. A shortened version contained only 19 questions relating specifically to participation in The Stretching Trial, and one question regarding future participation in Internet-based trials. (See Appendix 1 for the full set of survey items). The standard survey was made available to participants who completed 50% or more of the follow-up reports (6 or more reports) in The Stretching Trial, and the shortened survey was made available to other participants. The short version was provided in an attempt to encourage participants in The Stretching Trial to provide us with information about why they failed to participate fully and what researchers could do to improve follow-up rates.

The surveys contained both open and closed questions. Closed questions also contained a free text area for participants to provide more information if they wished. None of the questions were compulsory. The survey was pilot-tested by three participants of The Stretching Trial. Their results have been excluded from the analysis. The wording and structure of the survey was modified after the pilot.

### Analysis

Descriptive statistics were calculated for closed-ended questions. A comparison of the characteristics of The Stretching Trial participants who did participate in this survey and those who did not was done using a 2-sided *t* test (for age) and a χ^2^ test (for gender and follow-up weeks). We used SAS statistical software (version 9.2; SAS Inc, Cary, North Carolina) to calculate all statistics.

Content analysis was used to analyze open-ended responses [13]. A list of 106 codes were developed from the open ended responses about advantages and disadvantages of participation. After finalization, the complete list was then re-applied consistently to all of the data. This allowed us to determine which were the more or less frequently occurring codes. The codes were then grouped into 6 main categories. Each of the 6 categories had a ‘positive’ and ‘negative’ dimension. That is, that most advantages of participation had a ‘mirror’ disadvantage of participation: advantages and disadvantages could be conceptualized in pairs. These pairs, or trade-offs, will be presented and discussed below.

## Results

1249 participants completed The Stretching Trial in English. 1246 invitations to participate in the survey were emailed between May and October 2009. Data were collected between May and December 2009.

Ten email messages were undeliverable. 416 participants completed the survey, so the response rate was 33.4% (416/1246). Those who participated in this survey were slightly older (46.3 years v 41.2 years, p<0.0001) but similar in terms of gender (% male: 39.9 v 41.9, p=0.49) compared to those who did not participate (Table 1).

**Table 1:** Baseline demographics and response rates

	**Whole sample (n=1246)**	**Survey responders (n=416)**	**Survey non-responders (n=830)**	**Comparison between responders and non-responders**
**Age**				
mean (SD)	42.9 (13.1)	46.3 (13.1)	41.2 (12.8)	t_1244_=−6.59, p<0.0001
range	21-88	23-78	21-88	
median	42	45.5	39	
**Gender**				
male n (%)*	514 (41.3)	166 (39.9)	348 (41.9)	*X*_1_^2^ =0.468, p=0.49

*Percentages are percentage of group (column) totals.

While the response rate overall was 33%, response rates among participants offered the standard versus the short survey differed significantly (*X*_1_^2^ =237.13, p<0.0001). Among those offered the standard survey (that is, those who had completed 6 or more weeks in The Stretching Trial) 48.0% (398/830) completed the survey. However, only 18 of the 416 people offered the short survey (those who had completed less than 6 weeks in The Stretching Trial) completed it (4.3%). Therefore we present the results of the standard survey throughout. Where the responses differed between the standard and the short survey, results from the short survey are also presented.

### Participating in the stretching trial

Most participants became aware of the study via email (Table 2). Other methods included articles in newspapers, magazines or journals, and online search engines, websites or links.

**Table 2:** Participation in the Stretching Trial

**Recruitment**	**n (%)**
**How did you hear about the stretching trial? (n=397)**	
online (search engines, websites, links)	73 (18.4)
email	94 (23.7)
newspaper/magazine/journal	86 (21.7)
newsletter/flyer	6 (1.5)
news	2 (0.5)
radio	30 (7.6)
other	19 (4.8)
don’t know	29 (7.3)
person	58 (14.6)
**Would have participated in stretching trial if data was collected using:**	
phone (n=380)	130 (34.2)
written surveys (n=380)	174 (45.8)
face to face interviews if:	
accessible by public transport (n=368)	85 (23.1)
accessible by driving (n=370)	62 (16.8)
**Follow-up and reporting outcomes**	
**Time to complete weekly report (n=395)**	
≤5mins	310 (78.5)
6-10mins	77 (19.7)
11-20mins	8 (2.0)
**Was reporting a burden (n=397)**	
not at all	297 (74.8)
a small burden	100 (25.2)
a major burden	0 (0.0)
**Difficulty in accurately reporting outcome (n=393)**	
very difficult	8 (2.0)
somewhat difficult	77 (19.6)
a little difficult	137 (34.9)
not at all difficult	171 (43.5)
**Reporting truthfully (n=391)**	
always truthful	298 (76.2)
usually truthful	87 (22.3)
sometimes truthful	6 (1.5)
rarely truthful	0 (0.0)
**Consistently reporting outcomes (n=391)**	
very consistent	173 (44.3)
somewhat consistent	178 (45.5)
a little consistent	34 (8.7)
not at all consistent	6 (1.5)

Reasons given for participating in the trial fell into four main areas. Two areas were personal interest in the research question and outcome, and ease of participation:

Easy to participate as it was Internet-based. Also as a runner and researcher I was interested in the research question. (ID806)

The next was appreciation of the importance of research:

I am a health researcher and appreciate how important research is and how difficult it can be to recruit. (ID70)

Others participated for altruistic reasons:

I am a regular bicycle commuter, so exercise was part of my routine. Additionally, I am a healthcare provider (nurse) and wanted to know if stretching was a valid routine. Finally, I wanted to assist my fellow man. (ID 576)

Less than half of participants said they would have participated in The Stretching Trial had it been conducted using other means of data collection (postal, phone or in-person interviews). The most acceptable alternative form for these participants would have been a written survey (postal) (45.8%, 174/380) (Table 2).

The majority of respondents (78.5%, 310/395) completed their weekly outcome measurements online in less than 5 minutes, and felt that weekly reporting was not at all a burden (74.8%, 297/397) (Table 2). Among those who stated that the weekly reporting for 12 weeks was a small or major burden, this was generally attributed to the study being one of many tasks to complete in limited time. As one participant wrote: “Just the time effort and one more task you, however little, you should do in a busy week. (ID852)” A small number of participants reported that limited access to a computer and the Internet was a reason for this burden, with comments such as: “I would sometimes not get it completed immediately as I don’t always have access to a PC - then I would get reminders and have to think back on the previous week. (ID306)” When participants were asked what researchers could do to minimize burdens, they typically gave answers such as: “Not much, I knew what I was getting into, the reminders were great, though. (ID 576)”

Among those who completed the short survey (ie completed less than 6 weeks of follow-up in the trial), only 55% (6/18) completed their weekly outcome measurements in less than 5 minutes, and only 36% (5/18) felt that weekly reporting was not a burden at all.

Reasons given for poor follow-up by those who completed less than 10 weeks of The Stretching Trial (n=30; less than 6 weeks follow-up n=18, 6–9 weeks follow-up n=12) could be categorized into 3 main areas: time, change in personal circumstances, or specific problems with the study. For those for whom time was an issue, some said they were too busy to continue. Most of the participants who reported a change in circumstances reported sustaining an injury either between registering their interest and commencing the study, or reporting an injury unrelated to the lower-body which meant they were no longer exercising. Specific perceived problems with the study included technical issues, unhappiness with allocation, and not liking the questions asked.

A large proportion of participants experienced some difficulty in reporting outcomes accurately, with only 43.5% (171/393) experiencing no difficulty at all (Table 2). Of those that experienced some difficulty, the main concern was the subjective nature of the outcome measure:

It was difficult to grade a score out of 10 - although I am unable to suggest a better alternative … Plus the definition of soreness would have been different for everyone. (ID331)

Other reasons included difficulty selecting a suitable response from the limited response options available and the inability to provide more information:

There could have been more options, or the ability to write something like here. Might have provided you a little more information from the participants. I know I wanted to write things. (ID 141)

Most respondents said they reported truthfully each week (76.2%, 297/391)(Table 2). 76 participants elaborated in free text, with most emphasizing the gap between intention and execution: although participants attempted to report truthfully, they sometimes had difficulty remembering from the past week or felt the wording/format of the questionnaire did not allow them to report as truthfully as they would have liked to. A few participants asserted the importance of being truthful. Only five participants claimed to have knowingly misreported their exercise or stretching activities. Two of these participants said they wanted to report the ‘desirable’ answers for the benefit of themselves or the research.

How many people admit that they fudged a little! In the interests of science (and anonymity) I sometimes fudged a little and said that I exercised slightly more than I had (say 1 more session than usual) - it made me feel better about myself. (ID 331)

At times I wasn't performing all of the stretches (I was in the stretching group) or would not stretch before AND after exercise. (ID271)

It was hard for me not to stretch when it has played such a large part in my exercise routine. Also I noted that a few times I wanted what to report what I felt should be right rather than what was actually occurring. (ID86)

More people were willing to confess reporting levels of soreness from one week to the next inconsistently (Table 2), with only 44% of participants claiming to report outcomes ‘very consistently,’ and 99 participants commented about this. These accounts suggested some definitional confusion between ‘truthful’ reporting and ‘consistent’ reporting, as the two were attributed to similar reasons: the subjective nature of the outcome and insufficient opportunity to qualify answers.

### Participating in internet trials in general

#### Advantages and disadvantages of participating

As discussed in the methods section, the advantages and disadvantages of participation were captured in 106 codes. We were able to group these codes into 6 overarching categories. The codes and categories are presented in Table 3. After grouping the codes into categories we developed an original, interpretive finding: advantages and disadvantages could be conceptualized in pairs. Each of our six categories was a quality or characteristic of internet trials; each had a positive and negative dimension. The six categories were:

1. *Flexibility, convenience, connectedness and understanding for participants*

2. *Degree of burden and ease of use for participants*

3. *Security, Privacy and Confidentiality*

4. *Researcher experience, as perceived by the participants*

5. *Technical aspects of the research technology*

6. *Information quality*

**Table 3:** Advantages and disadvantages of participating in Internet RCTs

**Advantages (n=364)^**	**Disadvantages (n=339)^**
**1. flexibility, convenience, ability to control time and place of participation**	**1a. less flexibility, less interpersonal connectedness and communication,**
own time^A^	no personal contact^B^
convenient^A^	can only use email to f/u^C^
any time/flexible time to complete^B^	less motivation/less rewarding^C^
easily accessible^B^	no direct feedback^C^
no travel/convenient place^B^	no interaction between participants^C^
no appointments^B^	no discussion about how study is going^C^
access study from anywhere^B^	disjoint - not part of the research^C^
flexibility/allows for busy lives /fits into my schedule^B^	more personal benefits from conventional research^C^
accessible at all times^C^	no follow-up possibilities^C^
I have the choice to participate/find out about study^C^	can not deviate from questions/allow for special circumstances^B^
	prefer mix of methods^C^ no one to talk to incase of adverse events^C^
	**1b. difficulty in understanding/being understood**
	can't ask questions/can't get immediate answers^A^
	no advice about intervention or outcome reporting^B^
	mis-interpret/don't understand question/information^B^
	not enough detail in answers/fixed answers^B^
	difficult to convey message^C^
	unable to clarify/provide/ask for further details^C^
	computer literacy an issue for some^C^
**2. ease of use/burden**	**2. difficulty in use/burden**
easy^A^	a burden^C^
not time consuming^A^	too busy at work for emails^C^
not a burden/not a lot required/little effort to be involved^B^	need to check emails regularly^C^
no post or phone calls to worry about^C^	takes time^C^
frequently check emails/on Internet^C^	go away with no access can't participate^C^
user friendly^C^	time on computer^C^
typing is easier than writing^C^	
fun/ enjoyed participating^C^	
can receive the results^C^	
**3. Security, privacy and confidentiality**	**3. Security, privacy and confidentiality**
anonymity^B^	online security^C^
non-invasive^C^	invasive (over everyday workload) ^C^
lack of pressure^C^	genuine study or hoax^C^
can participate without others knowing^C^	
**4. participant perceived benefits to the researchers**	**4. participant perceived disadvantages to the researcher**
decreased cost^B^	easy for participants to drop out^C^
greater geographic reach^B^	participants can register twice^C^
quick results^C^	unaware who is participating^C^
more likely to continue for longer f/u^C^	limited research area^C^
faster data entry^C^	less controlled^C^
quick fixes of issues while study is underway ^C^	difficult for qualitative data^C^
researchers compile data accurately^C^	self selected participants^C^
fewer dropouts^C^	research may not be seen as significant as other forms^C^
blind person can participate^C^	those without computer/Internet access not able to participate^C^
quantitative^C^	
continuous analysis^C^	
more potential participants/large number of participants^C^	
many advantages for researchers^C^	
**5. technical aspects**	**5. technical aspects**
reminders^B^	forget to complete/need reminders^B^
could stop when busy and return to complete it later^C^	personal Internet issues/connections/need access to a computer^C^
can use multimedia to assist ^C^	emails may be filtered as junk/spam^C^
unable to skip questions (may assist with data quality) ^C^	could result in too many email requests for more^C^
	difficult to make changes if mistake made^C^
	online distractions while participating^C^
**6. positive effect on information quality**	**6. negative effect on information quality**
time to think^C^	honesty may be compromised^B^
more honest results^C^	accuracy may be compromised^B^
not influenced by the interviewer^C^	subjective measures^B^
data collection is consistent^C^	little thought if rushed^C^
more accurate results^C^	sensitive info not disclosed^C^
was relaxed when answering questions/no stress^C^	need to recall answers^C^
easily correct mistakes^C^	not as serious as other forms^C^
no answers received^C^	lower data quality^C^
not judged^C^	results not as valid as other forms^C^
better compliance^C^	self report^C^
forces to answer question specifically asked^C^	superficial form of research^C^
aware of answers supplied/can see questions and answers^C^	
**7. no advantages**^C^	**7. no disadvantages**^B^
**8. paper wastage**^C^	
**9. wouldn't participate otherwise**^C^	

^more than one response could be provided

A: most frequently reported (n>65+);

B: frequently reported (15≤n≤64);

C: least frequently reported (1≤n≤14)

Because each of these six categories had a positive (advantageous) and negative (disadvantageous) dimension, we concluded that the advantages and disadvantages of internet trials could be conceived of as trade-offs. Each advantage of internet research has a ‘mirror’ or ‘twin’ disadvantage. We note that this is our interpretive analysis across all of the data: while some individuals discussed both negative and the positive dimensions of a given category, others might emphasise one or the other. Overall, more advantages were noted by participants, consistent with their preference for this mode of research over others. Participant perceived advantages and disadvantages are shown in Table 3.

##### The benefit of flexibility and convenience versus the disadvantage of lack of connectedness and understanding

The most dominant perceived advantage of Internet-based research was the flexibility and convenience of participating. However, this was offset by the two most dominant disadvantages: i) a lack of interpersonal connectedness and communication and ii) difficulty in understanding/being understood.

Participants were appreciative of the ability to complete the study in their own time and at any time of the day. There were no requirements to be at a particular place, on a particular day at a particular time. This allowed participants to fit the study into and around their already busy lives.

I can choose the time of the day I'll answer the questions, and the environment is familiar (I do it at home or work, and not a hospital or clinic). (ID45)

People also valued being able to have the choice to participate directly in research that they identified as being of interest to them, rather than being dependent upon doctors or other health care professionals to inform them about research participation opportunities.

However, participants did miss the personal contact with both researchers and other participants in the study. They often lacked motivation to continue, finding the online research experience less rewarding than other forms of research. Participants felt that Internet-based research limited their ability to ask questions of the researchers and obtain information regarding the intervention and outcome reporting. They found it difficult to convey information to researchers due to the restricted space in which they could provide in the answers. The inability to deviate from the questions asked, and the lack of ability to take into account individual circumstances were also frequently reported as a disadvantages. It was also noted that some people would face difficulties in communicating or participating if they were not particularly computer literate.

The lack of feedback. If my response had been given in person or over the phone, there would probably have been some chat about how the survey was going. Because of the lack of this, I never really felt part of the research. (ID47)

There were five other key trade-offs identified. In regard to each, online research was considered advantageous in some respects and disadvantageous in others. Participants differed on whether online research, relative to offline research, was: 1) more or less burdensome; 2) more or less secure, private and confidential; 3) better or worse for researchers; 4) assisted or hampered by technical aspects of the Internet; and 5) likely to produce information of better or worse quality. We discuss each of these below.

##### Degree of burden and ease of use

Consistent with comments about the experience of participating in The Stretching Trial (reported above) most people responded that the Internet is easy to use, and that participation was not much of a burden. Most often, this was attributed to the small time commitment required, or the ease of use, ‘user friendliness’, or even ‘fun’ of it. An online study may be easily be integrated into participants’ daily tasks because they are already frequent Internet or email users. An online study means no post or telephone calls and typing which is easier than writing with a pen. Far fewer people identified participating in Internet research as burdensome but when they did, computer access was identified as the main problem. This could include lack of access (for example, while on holidays), or being too busy at work to check email.

##### Security, privacy and confidentiality

Participating in Internet-based research provided some participants with an increased feeling of anonymity and privacy. The non-invasive nature and a lack of pressure from the researchers to either answer in a particular manner or to continue with the study were also noted as advantages of Internet research. The ability to participate in research without disclosing involvement to others was considered particularly important when the topic was of a sensitive nature. However, these advantages were contrasted against confidentially and online security issues. These were particularly important if personal information was collected. Some participants noted that the study or the researchers could be illegitimate and attempts could be made to deceive people and elicit personal information from them fraudulently.

The disadvantage would be the fact that I may not be able to tell whether the study was genuinely conducted by the University or just a hoax. But I felt that you guys did a good job in identifying yourselves as a legitimate group conducting a genuine research study and that eased my mind on the matter. As you may know, the Internet has a lot of evil people trying to get access to personal information via similar methods. (ID208)

When prompted to reflect on this issue, 39.0% (146/374) of participants reporting some degree of discomfort entering health information over the Internet, and only 51.2% (168/328) said they would supply sensitive health information over the Internet when they were also providing identifying information such as email addresses, name and postal addresses, or date of birth.

##### Participant-perceived benefits and disadvantages to the researcher

Although participants were asked what they thought the advantages and disadvantages were to participants, several listed reasons that would apply to the researchers. These included the decreased cost of conducting such research and also greater geographic reach which would result in greater potential to recruit larger numbers of participants. Participants also reasoned that data entry would be quicker and more accurate for researchers, and the results would be available sooner. However, it was recognised that the researchers might have less control over participants and may not be fully aware of who is participating in the research. It was also identified that there is the possibility that researchers are excluding people without computers or Internet access. There is also the possibility of participants registering multiple times. Participants said that Internet research may only be appropriate for some research topics.

I just think it's neat! Being able to use the Internet for medical surveys allows people all over the world to participate in studies that they would otherwise not be able to, especially when the surveys do not require extensive medical testing or histories. It's a small world after all. (ID242)

Organiser does not know who is really taking part - I could be 15 year old boy or 80 year old woman … (am neither!). (ID231)

Some people may be excluded as they don't have access to the Internet eg some older people, deprived populations. (ID390).

##### Technical aspects

Some participants claimed that because reminders to complete outcome measures arrived as emails, they could be forgotten easily unless acted upon immediately. Conversely, participants greatly appreciated the email reminder system, and saw this as a positive aspect of Internet-based research. Participants liked being able to easily save the data they had entered and return to it later if they were interrupted. However, it was also noted that it was easy to become distracted while on the Internet by other Internet applications. This meant participants may not be completely attentive during their participation in either the intervention (if delivered online) or outcome measurement. Participants suggested that multi-media presentation may assist with the delivery of the intervention or outcome measurement and enhance understanding of what is required. Internet connections were problematic for some, as were potential issues with participants using different web-browsers or operating systems than the systems the trial website was developed for. The potential to receive junk emails, or an increase in emailed requests to participate in future trials was also a concern for a few participants.

##### Information quality

Many participants noted issues surrounding the quality of information collected using the Internet. Some stated that they were more likely to be honest and accurate, as they had more time to think about their answers and were not influenced by the perceived judgment of the researcher, as they had experienced in face to face research. Conversely, some argued that it may be easier in Internet research to ‘bend the truth’: many pointed out that this is not what they had done or would do, but imagined that others might do it. Others commented on the subjective, self-report nature of data collection and that the research conducted may be fairly superficial. Together, these criticisms suggested that Internet research may not be perceived as being as rigorous as other forms of research, and that the results may not be perceived as being as valid.

When prompted to reflect upon this issue, only a small proportion of respondents (4.3%, 16/373) said that they would make a false statement to appear eligible to participate in a trial (age, gender, ethnic background and health status were the eligibility criteria considered). This would generally only occur when participants thought they were close to the eligibility criteria, or those criteria were not important. These results varied little over modes of data collection (telephone, personal interview, Internet and written/postal).

A substantial proportion of respondents (27.4%, 99/361) said they would consider making a false statement about sensitive health information in medical trials, with more participants (54.7%) likely to make a false statement in face to face interviews compared to over the Internet (30.1%). Of the four modes of data collection enquired about (Internet, postal, phone and in-person interviews), those who considered themselves more likely to truthfully report sensitive health information via personal interviews said they were least likely to report truthfully via the Internet. Conversely, those that felt the Internet was the best method to obtain truthful information, felt less likely to be truthful in a personal interview (Table 4).

**Table 4:** Honesty in Internet trials in comparison with other modes of data collection

**Provide false information regarding sensitive health issues (any mode of data collection) (n=361)**	
Would never make a false statement	269 (72.6)
Could possibly make a false statement	99 (27.4)
**Mode of data collection most likely to make a false statement (n=86)**	
personal interview	48 (54.7)
mean rank for Internet~	3.65
median rank for Internet~	4
Internet	26 (30.2)
mean rank for personal interview~	3.88
median rank for personal interview~	4

~1=most likely to make false statement, 4=least likely to make false statement.

### Compulsory questions

In Internet-based research it is possible to make questions compulsory by requiring that all questions are completed prior to continuing to the next ‘page’ of questions. Participants’ opinions of this feature varied. The majority of participants were happy with compulsory questions provided i) that this was a feature used to check the person hadn’t accidently missed a question and they had the ability to ‘opt-out’ of the question with options such as “N/A” or “I’d prefer not to answer this question” ii) there were sufficient options to allow the participants to provide an answer that was correct, or ii) there was a free text area. If a suitable answer was not available, participants imagined that they would consider picking a false answer to continue, or simply stop participating.

### Participation in future internet trials

The majority of participants (69.5%, 271/390) said they would prefer to participate in Internet-based research over other modes of data collection (postal, phone and in-person interviews) in the future. 20.5% (80/390) had no preference.

Less than 1% (1/379) of respondents said they would not participate in future Internet-based randomised controlled trials.

In contrast, of those that completed the short survey, slightly fewer said they would prefer an Internet-based study (10/18, 56%), with more participants expressing a preference for postal (3/18, 17%) or face to face (2/18, 11%) data collection compared to those who completed the standard survey (5/390, 1.3% and 9/390, 2.3% respectively).

## Discussion

### Summary of results

To our knowledge, this is the first study to evaluate participants’ experiences and perceptions of participating in Internet-based RCTs. Participants’ comments and reflections on their participation of participating in a fully online trial, The Stretching Trial, were positive. Less than half of participants said they would have participated in the trial had it been conducted using other means of data collection. Weekly reporting took participants less than 5 minutes, and was not seen as a burden. In contrast, the majority of those that completed less than 50% of follow-up weeks spent more than 5 minutes completing their weekly report, and experienced some burden completing these reports.

Our central, interpretive and most original finding in this study was that elements of participating in Internet-based trials have both positive and negative dimensions. Most of the advantages of participation had a ‘mirror’ disadvantage of participation and could be conceptualized in pairs, or as trade-offs. The main trade-off was between flexibility and convenience – a perceived benefit – and lack of a feeling of connectedness and understanding – a perceived disadvantage. Other trade-offs identified included ease or difficulty in use of the Internet; security, privacy and confidentiality issues; perceived benefits and disadvantages for researchers; technical aspects of using the Internet; and the impact of internet data collection on information quality. Overall, more advantages were noted by participants, consistent with their preference for this mode of research over others. The majority of participants would prefer to participate in Internet-based research compared to other modes of data collection in the future.

### Strengths and limitations of this study

This is the first study of its kind, and our findings are important because of the increasing use of the Internet to conduct randomized trials. This information can be used to inform future study design. Although several studies have compared follow-up rates, completeness of data, and reliability of surveys using the Internet [14-18], and others provide experiences of Internet randomised trials from the researchers perceptive [19], none have reported on the participants’ perspective.

As participants in this study came from a range of English speaking countries around the world, the global nature of this study provides researchers around the world with valuable information.

All participants involved in this survey were recent participants of a fully online RCT. They all had Internet access and were prepared to participate in a 12 week trial. Their thoughts and experiences may not be representative of the entire population, particularly those who choose not to participate in medical research conducted via the Internet. Furthermore only 33.4% of those invited to participate completed the survey. These participants are likely to be highly motivated to participate in research, and have already participated in online research. Participants were reporting upon their experiences of participating in a fully online trial. As such, the results may not apply to other trials which use the Internet for only part of the trial. Participants were asked how they thought they would respond in certain situations in the future. For example, they were asked if they would participate in future Internet-based research. In general, however, people are poor at predicting future behavior [20], and their answers may have been vulnerable to social acceptability biases. This survey was only conducted via the Internet. Alternative forms of contact was not possible as no personal contact details (other than email addresses) were collected during the RCT. Participants who may have had a negative experience using the Internet for research may have been less inclined to participate in our survey. Future surveys may benefit from using various modes of data collection (eg. postal, phone and internet). Despite these limitations, our sample does represent a proportion of the population who are interested and willing to participate in Internet-based medical research, and therefore their answers are relevant to the design and management of future Internet-based trials.

### Data collection

Many participants were frustrated by the limited ability to qualify individual responses in Internet-based trials. Allowing participants an option of providing ‘free text’ in online data collection would overcome this issue, however, in large trials, the inclusion of such a ‘free text’ area may be unfeasible, as it may be impractical to analyze large sets of unstructured data. In smaller trials provision of an opportunity to enter free text should be considered. In certain circumstances, the provision of “not applicable” or “none of the above” may be sufficient. Some of the issues that participants noted with data collection (for example self report and subjective reporting) are not limited to data collection via the Internet, and apply to all modes of trial conduct. In the case of Internet-based trials, self report is unavoidable, and subjective reporting is likely to occur in almost all instances, whereas researchers conducting trials using other, more conventional modes can design their trials to avoid these problems if desired.

### Truthfulness of disclosure

In this study, there appeared to be two groups of participants with different preferences regarding the disclosure of sensitive health information. One group preferred the anonymity of the Internet, and the other preferred the personal contact and trust of the researcher. In reality, it would be difficult to distinguish between these groups of people prior to participating in research, so it is important to recognise this limitation in any form of research in which sensitive issues are being discussed.

It is interesting to consider whether having a researcher present may influence how truthfully people report. Some participants felt it necessary to affirm how important they thought being truthful was, and only a few people found it possible to confess that they had been less than truthful. Interestingly, more participants were prepared to confess to ’inconsistencies’ in reporting than untruthful reporting. We need to consider the possibility that participants had difficulty in defining, or distinguishing the difference between ‘consistency’ and ‘truthfulness’. Many participants reported the same reasons for inconsistency and untruthfulness. It is easier, and more socially acceptable to consider these issues as obstacles when reporting ‘consistency’ rather than ‘truthfulness’. Researchers conducting data-collection via the Internet could consider using multimedia in an attempt to increase truthfulness. This could include providing brief instructions that mimic those given in face to face data collection.

### Significance of this research

The main methodological limitation of Internet trials is high rates of loss to follow-up [10]. The results of this study provide researchers with an insight into the participants’ perspectives on Internet trials. Understanding the advantages and disadvantages of Internet-based trials from the perspective of participants, and designing studies accordingly to accentuate the advantages and minimise the disadvantages to the participant may serve to increase follow-up rates within trials.

Participants perceive there to be both advantages and disadvantages to participating in Internet-based research. Overall, the advantages outweigh the disadvantages, and the participants in this study would prefer to be involved in Internet-based research in the future as opposed to other modes of data collection. Findings thus appear to support ongoing development of Internet trials and methodological research to improve them. Many people had issues with privacy and confidentiality and researchers need to be aware of these concerns when setting up Internet trials.

### Future research in this area

We suggest future research testing our categories which were created to classify the advantages and disadvantages reported by the participants, and their positive and negative dimensions, in order to determine prevalence of agreement, as well as the preferences and prevalence in different population groups.

We also recommend further investigation into the relationship between the mode in which trials are conducted and the truthfulness of disclosure. An exploration into the reasons why people are more or less likely to report truthfully when participating in Internet-based trials, and the implications this has upon the trial results is warranted.

## Conclusion

Ultimately, participants in our survey would prefer to participate in Internet-based trials in the future compared to other ways of conducing trials. From the participants’ perspective, participating in Internet-based trials involves trade-offs. The central trade-off is between flexibility and convenience – a perceived benefit – and lack of connectedness and understanding – a perceived disadvantage. Strategies to maintain the convenience of the Internet while increasing opportunities for participants to feel supported, well-informed and well-understood would seem likely to increase the acceptability of Internet-based trials.

## Appendix 1: Survey Items

### Standard survey

1. How did you first hear about the stretching study? (open ended)

2. What was it that made you want to participate? (open ended)

3. Thinking about the weekly emails and reminders you were sent, and completing your weekly reports:

a. How long did each weekly report take to complete on average?

 ● □ 5 minutes or less

 ● □ between 6-10mins

 ● □ between 11–20 mins

 ● □ 21-30mins

 ● □ more than 30mins

 ● □ Other:_________________________

b. Did the weekly reporting become a burden?

 ● It was not a burden at all **(skip to Question 3c)**

 ● It was a small burden

 ● It was a major burden

(if answered small, or major)

1. Describe how it was a burden

2. What could we have done to lessen the burden?

a. How difficult do you think it was to provide accurate information about your muscle soreness and/or injuries?

 ● □ Very difficult

 ● □ Somewhat difficult

 ● □ A little difficult

 ● □ Not at all difficult

 ● □ Comments:____________________________

(**If answered very, somewhat or a little)**

Please explain what it was that you found difficult (open ended)

a. Do you feel you reported truthfully each week?

 ● □ Always truthful

 ● □ Usually truthful

 ● □ Sometimes truthful

 ● □ Rarely truthful

 ● □ Comments:_________________________

b. Did you feel you reported consistently each week? (for example, you had a certain level of soreness during week one, and again in week 3, were you able to report the same levels in the weekly reports)

 ● □ Very consistent

 ● □ Somewhat consistent

 ● □ A little consistent

 ● □ Not at all consistent

 ● □ Comments:_________________________

c. Do you have any other thoughts about the processes we used to collect information from you?

d. Would you have participated in this study if you had been required to complete your weekly reports:

1. over the phone at a set time each week? Y/N

2. in a written survey that was sent to you each week, which you were required to complete and send back? Y/N

3. by attending a clinic to meet with investigators at a set time each week if the clinic was:

a. accessible by public transport? Y/N –

**if yes,** The longest amount of time I would spend on public transport to get to and from the clinic would be: ______

b. accessible only by driving? Y/N –

**if yes,** The longest amount of time I would spend on public transport to get to and from the clinic would be: ______

Comments:_______________________

4. Next time you are considering participating in a research study would you prefer to participate in a study conducted (select only 1)

 ● □ On the internet

 ● □ Over the phone

 ● □ in a written survey which would be posted each week

 ● □ by attending a nearby clinic

 ● □ It wouldn’t make any difference to me how the study was conducted I would participate if I could

 ● □ Other

Comments:________________________________

Other past research

5. Have you participated in any other medical research trials? Y/N

If yes

h. What were these studies?

i. A one-off phone survey Y/N

ii. A one-off interview in person Y/N

iii. A one-off written questionnaire Y/N

iv. A one-off internet survey Y/N

v. Longer studies that required you repeatedly provide information over several days/weeks/months Y/N

1. If yes – how long did your involvement in this study last for? (From the start of yoru first involvement to the end of you last involvement, in weeks or months or years)?

2. What were you required to do during the study?

Internet trials / future research

Conducting research on the internet is a fairly recent development in medical research. In the past most medical research that involves humans, collecting information was carried out in person, or over the phone, or with postal surveys.

6. As a participant in medical research (having participated in the stretching trial) - what do you see are the **advantages** for you, when participating in medical research on the internet compared to participating in conventional research (research conducted in person, over the phone or in written surveys)

7. What do you see are the **disadvantages** for you, when participating in medical research on the internet compared to participating in conventional research (research conducted in person, over the phone or in written surveys)

8. Do you think you would participate in other medical trials that are conducted entirely on the internet?

 ● Yes,

 ● Maybe – it would depend on:_________,

 ● No.

Comments: _______________________

9. One feature of conducting trials on the internet is the ability for researchers to set the internet page so that you MUST answer all questions before continuing on to the next page. This differs from written surveys where participants can just skip questions by leaving them blank.

How would you feel if you participated in an online trial and it was a requirement that you answered all questions before moving onto the next page?

We want to explore the quality of information that people provide on the internet as more researchers are using the internet to conduct medical research, including research that might be about sensitive health information.

Thinking about medical researchers collecting information about you **as part of a research study conducted on the internet:**

10. How uncomfortable do you feel about entering information about your health on the internet?

 ● Very uncomfortable

 ● Somewhat uncomfortable

 ● A little uncomfortable

 ● Not at all uncomfortable

 ● Comments:_________________________

11. Would you disclose sensitive health information about yourself (such as information about drug or alcohol use, sexual health information) on the internet if:

a. you had **not provided** researchers with personal details such as your name, postal address, or date of birth? Y / N

Comment:__________________________________

b. you **had provided** some form of identifying information (eg email address, name/postal address, date of birth)? Y/N

Comment:__________________________________

Thinking about medical research in general (research conducted either in person, over the phone, written questionnaires, over the internet or any combination of the above)

12. Do you think you would provide false information about your sensitive medical information (such as information about your drug or alcohol use, or sexual health information):

(select only 1)

 ● □ I would never make a false statement about sensitive medical information while participating in medical research **Go to Question 14**

 ● □ I could possibly make a false statement about sensitive medical information while participating in medical research **Go to Q13**

15. Would you be more likely to make false statements regarding sensitive medical information over the internet, compared to a telephone interview, written questionnaire or in person?

(rank these in order of method most likely to make false statements – if you feel equally about 2 or more of the methods – please indicate this in the comments)

I’m more likely to make false statements about sensitive medical information:

Comments/Why

1. over the internet

2. in a telephone interview

3. in a written questionnaire

4. in a personal interview

14. All forms of medical research have a list of characteristics that participants must meet in order to participate (these are called eligibility criteria). These include limiting participants to a particular age range, ethnic background, gender, and health status.

Thinking about medical research in general (research conducted either in person, over the phone, written questionnaires, over the internet or any combination of the above)

Imagine you have heard of a study that you would like to participate in, however, you don’t meet one of the eligibility criteria, for example you are not the right age group for the study:

Would you make a false statement regarding your eligibility in order to participate?

 ● □ I would never make a false statement regarding my eligibility **Survey finish**

 ● □ I would consider making a false statement regarding my eligibility **Continue to Q15**

15. You have indicated that you would consider making a false statement regarding eligibility into a study that you are interested in participating in…

(**question a, b, c and d to be presented in random order)**

a. In this study information is collected on the internet – you will never speak with or meet the researchers, if you wish to contact the researchers, you need to send them an email:

In order to participate in the study, would you make a false statement about your:

i. Age Y/N – comments:

ii. Gender Y/N – comments:

iii. Ethnic background Y/N – comments:

iv. Heath status Y/N – comments:

b. In this study information is collected by mail – you will receive information in the mail, you need to sign a form, send it back, and will receive all study materials in the mail, and will complete written questionnaires which you will send back to the researchers. If you have any questions you can email, write a letter, or telephone the researchers.

In order to participate in the study, would you make a false statement about your:

v. Age Y/N – comments:

vi. Gender Y/N – comments:

vii. Ethnic background Y/N – comments:

viii. Heath status Y/N – comments:

c. In this study information is collected by telephone – you will receive information about the study, and you will receive a phone call about a study. If you participate, you will be sent materials in the mail, but will be interviewed over the phone:

In order to participate in the study, would you make a false statement about your:

i. Age Y/N – comments:

ii. Gender Y/N – comments:

iii. Ethnic background Y/N – comments:

iv. Heath status Y/N – comments:

d. In this study information is collected in a face to face interview – you will receive information about the study, in order to participate, you need to visit the researchers and complete interviews in person.

In order to participate in the study, would you make a false statement about your:

i. Age Y/N – comments:

ii. Gender Y/N – comments:

iii. Ethnic background Y/N – comments:

iv. Heath status Y/N – comments:

Shortened Survey

1. How did you first hear about the stretching study? (open ended)

2. What was it that made you want to participate? (open ended)

3. Of the 12 weeks followup, you completed less than 6 weekly reports. We are interested finding out why you did not complete the full 12 weeks. Please list all reasons and describe in as much detail as possible.

4. Thinking about the weekly emails and reminders you were sent, and completing your weekly reports:

a. How long did each weekly report take to complete on average?

 ● □ 5 minutes or less

 ● □ between 6-10mins

 ● □ between 11–20 mins

 ● □ 21-30mins

 ● □ more than 30mins

 ● □ Other:_______________________________

b. For the weekly reports that you did complete, did the weekly reporting become a burden?

 ● It was not a burden at all **(skip to Question 4c)**

 ● It was a small burden

 ● It was a major burden

(if answered small, or major)

1. Describe how it was a burden

2. What could we have done to lessen the burden?

c. How difficult do you think it was to provide accurate information about your muscle soreness and/or injuries?

 ● Very difficult

 ● Somewhat difficult

 ● A little difficult

 ● Not at all difficult

 ● Comments:_______________________________

d. Do you feel you reported truthfully each week?

 ● Very truthful

 ● Somewhat truthful

 ● A little truthful

 ● Not at all truthful

 ● Comments:_________________________

e. Did you feel you reported consistently each week? (for example, you had a certain level of soreness during week one, and again in week 3, were you able to report the same levels in the weekly reports)

 ● Very consistent

 ● Somewhat consistent

 ● A little consistent

 ● Not at all consistent

 ● Comments:_________________________

f. Do you have any other thoughts about the processes we used to collect information from you?

g. Would you have participated in this study if you had been required to complete your weekly reports:

1. Over the phone at a set time each week? Y/N

2. With a written survey that was sent to you each week, which you were required to complete and send back? Y/N

3. Attending a clinic to meet with investigators at a set time each week if the clinic was:

a. accessible by public transport? Y/N –

**if yes,** The longest amount of time I would spend on public transport to get to and from the clinic would be: ______

b. accessible only by driving? Y/N –

**if yes,** The longest amount of time I would spend on public transport to get to and from the clinic would be: ______

Comments:________________________________

5. Next time you are considering participating in a research study would you prefer to participate in a study conducted (select only 1)

 ● On the internet

 ● Over the phone

 ● Written Survey which would be posted each week

 ● Visiting a nearby clinic

 ● It wouldn’t make any difference to me how the study was conducted I would participate if I could

 ● I would never participate in a research study again

Comments:________________________________

## Competing interests

All authors declare that they have no competing interests.

## Authors’ contributions

EM and AB were responsible for the conception, design, analysis and interpretation of data, GJ contributed to the conception and design of the study, SC contributed to the analysis and interpretation of data. EM drafted the manuscript. All authors revised it critically for important intellectual content, and gave final approval of the version to be published. EM is guarantor for the study. All authors had full access to all of the data and can take responsibility for the integrity of the data and the accuracy of the data analysis. All authors read and approved the final manuscript.

## Sponsors

EM was supported by an NHMRC PhD scholarship. The funding body had no role in any aspect of this study.

## Pre-publication history

The pre-publication history for this paper can be accessed here:

http://www.biomedcentral.com/1471-2288/12/162/prepub
